# Neuropathic pain appears to be the main symptom associated with higher disease burden and lower pain alleviation in degenerative lumbar disease fusion patients^☆^

**DOI:** 10.1016/j.bas.2025.104224

**Published:** 2025-02-25

**Authors:** Alexander Cristea, Bart F.J. Heijnen, Seung Won Park, Aleksandr Krutko, Carlos Santos, Wolfgang Senker, Vasileios Arzoglou, Paulo Pereira

**Affiliations:** a Medtronic Cranial and Spinal Technologies, Bakken Research Center, the Netherlands; b Chung-Ang University Hospital, South Korea; c Scientific Research Institute of Traumatology and Orthopedics, Russia; d Hospital Universitario Marques de Valdecilla, Santander, Spain; e Kepler Universitätsklinik Linz, Austria; f Hull and East Yorkshire Hospitals NHS Trust, United Kingdom; g Centro Hospitalar Universitário São João, Faculty of Medicine, University of Porto, Portugal

**Keywords:** Degenerative lumbar disease, Lumbar interbody fusion, Neuropathic pain, Persistent pain after spine surgery

## Abstract

**Introduction:**

The role of neuropathic pain (NP) in persisting pain after degenerative lumbar disease (DLD) fusion surgery appears to be underrecognized and undertreated.

**Research question:**

This study assessed NP in DLD patients before and after lumbar interbody fusion (LIF) surgery, and the NP-related burden of disease up to 12-months post-op.

**Materials and methods:**

Within a prospective, multi-center, data-monitored study, a sub-cohort of 146 DLD patients underwent LIF. NP was assessed pre-op and 3-months post-op with a validated Douleur Neuropathique-4 questionnaire. Outcomes were analyzed based on NP occurrence at baseline and post-op. Medication use, back-pain, leg-pain, Oswestry Disability Index (ODI), and quality of life (QoL) were determined pre-op, 3-months and 12-months post-op. Fusion success was evaluated via x-ray and/or CT-scan. Changes were analyzed using paired t-tests and ANCOVA to test for group differences.

**Results:**

NP was present pre-op in 51% of the DLD patients associated with higher back- and leg-pain, and lower QoL. LIF resulted in significant pain relief and improved QoL for all patients. Patients presenting NP post-op had significantly lower back- and leg-pain relief, ODI and QoL up to 1-year post-op. Opioid consumption was higher in the NP group, whereas DM and PVD occurrence, and fusion rates were similar.

**Discussion and conclusion:**

NP occurred frequently in DLD patients, both before and after spine fusion surgery. Patients with post-operative NP reveal a significant association between NP, lower pain alleviation and higher disease burden up to 12-months post-op, despite higher opioid consumption. NP occurred independently of DM, PVD and fusion success.

## Highlights

• Neuropathic pain in degenerative spine patients is underrecognized and undertreated.
• Determining neuropathic pain at pre-op AND post-op are both important.
• Pre-op neuropathic pain can be a predictor of persisting post-operative pain.
• Post-op neuropathic pain relates to lesser outcomes after spine fusion surgery.

## 1 Introduction

Low back pain is one of the most common health problems resulting in disability among older adults (Wong et al., 2017). Persistent pain affects more than one-in-five lumbar surgery patients and accounts for substantial long-term healthcare costs (Weir et al., 2020). Early recovery, statistically significant and clinically meaningful benefits in pain alleviation according to the visual analogue scale scores for low back pain (VAS-LBP) and leg pain (VAS-LP), and improved quality of life (QoL), were reported at 4 weeks after one- or two-level lumbar interbody fusion (LIF) in patients with degenerative lumbar disease (DLD) (Pereira et al., 2015a, 2023b). The early improvements in VAS-LP, VAS-LBP and QoL were maintained and improved at 12 months post-surgery (Franke et al., 2016).

In the MASTERS-D (NCT01143324) study, clinical success in reducing VAS-LP and VAS-LBP after LIF was defined as a clinically important difference (MCID) of at least 30%, or a change equal or superior to 1.5 points on a VAS scale from baseline (Asher et al., 2020). Clinical success in reducing back pain was achieved at two days after surgery in more than 46% of the patients, which increased to more than 70% by one year. Similarly, clinical success in reducing leg pain after LIF was achieved by 69% of patients at two days after surgery, increasing to 73% at one year (Franke et al., 2016). These results raised the question regarding the underlying causes for the discrepancy between high fusion success rate (90% at 1 year across all patient groups) and the lower rate of patients with pain alleviation.

Earlier reports of patients experiencing persistent pain after spine surgery (PPSS) ranged from 3 to 34% at 6–24 months post-surgery, and 5–36% at more than 2 years follow up (Parker et al., 2015; Strömqvist et al., 2014). The condition of persistent or recurrent leg and/or low back pain after one or more spine surgical interventions has often been called failed back surgery syndrome (FBSS), a condition associated with great healthcare utilization and costs (Petraglia et al., 2015). The designation has been criticized for implying the causative role of surgery in the persistent pain condition (Vleggeert-Lankamp et al., 2013), and for not indicating any predictive factors (Assaker and Zairi, 2015). Complex etiology of pain in spine conditions, including neuropathic pain (NP), heterogeneity of patient population and limited evidence from clinical studies were identified as factors responsible for inducing large practice variation in treating PPSS (Tronnier et al., 2019). The difficulty lies however in the predication and/or identification of patients at risk for limited pain relief after LIF.

The present study specifically investigates the occurrence of NP before and at 3 months after fusion surgery in adult DLD patients suffering from chronic leg and/or low back pain. In addition, we report on the impact of NP on pain levels, QoL and opioid and non-opioid pain medication use in these spine patients up to 12 months after surgery.

## 2 Methods

### 2.1 Study design

Within a prospective, multi-center, data-monitored, cohort study MASTERS-D 2 (NCT02617563) (Pereira et al., 2023b), a number of 365 DLD patients diagnosed with spondylolisthesis and/or stenosis, indicated for a single or double level lumbar interbody fusion procedure were prospectively enrolled between January 2016 and June 2019 at 26 sites across Europe, Asia, and Latin America (Pereira et al., 2023b). All patients received minimally invasive LIF (for more details see Pereira et al. (2023b)) and were followed-up according to standard of care (SOC) practice (Oberman, 2017). Informed consent was given by patients prior to any study-related procedure.

The study adhered to the Declaration of Helsinki (2013) and applicable local laws. Furthermore, if required by local regulations, ethics approval was obtained prior to patient recruitment and administration of any study procedure.

### 2.2 Neuropathic pain

We used the validated Douleur Neuropathique 4 questionnaire (DN4) to assess the presence of a NP component preoperatively (baseline) and at 3-months after spine fusion surgery (Hallstrom and Norrbrink, 2011).

The DN4 was introduced into a study that was already enrolling for minimally invasive LIF. Hence, not all patients could be administered the NP questionnaire at baseline and/or 3-months follow-up. DN4 data was collected prospectively, no retrospective data collection was done in patients that were already enrolled.

A DN4 score equal or above 4 out of 10 was considered as indicative of a NP component (Hallstrom and Norrbrink, 2011), when present in either the leg, the back or in both locations. NP was considered absent when DN4 score was below 4 in both locations. The same assessment and considerations were applied 3 months after the surgery. Only patients that could be assessed both at baseline and at 3 months post-surgery were included in the analyses.

We did not administer the DN4 for the leg or back when the patient reported absence of pain (i.e. VAS score of zero) for the leg or back. We considered the patient as not having NP in that location. Translated and validated versions of the DN4 were used as relevant per site.

### 2.3 Patient reported outcomes

Visual Analogue Scale (VAS), Oswestry Disability Index (ODI), and EuroQoL five-dimensions health-related quality of life index (EQ-5D) questionnaires, were administered prior to, at 3-months, and at 12-months after the LIF procedure. Validated VAS-scores (range: 0–10) were used to assess chronic leg-(VAS-LP) and low back pain (VAS-LBP). The ODI is a widely used, cross validated, self-administered questionnaire measuring disability associated with lower back pain (Fairbank and Pynsent, 2000). Translated and validated version of the EQ-5D-3L (hereafter referred to as EQ-5D) (Rabin and de Charro, 2001) questionnaire was used to assess health-related QoL; the index score was used for analyses (range: 0–1).

### 2.4 Clinical and surgical outcomes

Clinical parameters included general patient demographics, diabetes mellitus (DM) and peripheral vascular disease (PVD) co-morbidities, main reason for fusion surgery, patient reported outcomes and duration of pain before surgery. Lumbar fusion was assessed per SOC using x-ray and/or CT scan (Pereira et al., 2023b). Medication use questionnaires were administered prior to, at 3-months, and at 12-months after the LIF procedure.

### 2.5 Medication use

Opioid and non-opioid analgesic medication was recorded according to the World Health Organization's classification. We collected this information at pre-op, 3-months and 12-months after the fusion surgery. Frequency of medication use was collected without registering the actual dosage.

### 2.6 Statistical analyses

Results for continuous data are presented as mean and standard deviation (SD). The VAS-LBP, VAS-LP and EQ-5D index scores were analyzed using SAS software (Version 9.4 of the SAS System) to conduct Paired t-tests for changes at 3-months, and 12-months from baseline. ANCOVA analyses were used to test for group differences. The data that support the findings of this study are available on request from the corresponding author. The raw data are not publicly available to protect the privacy of the research participants.

## 3 Results

First, the results focus on the occurrence of NP in our study population, both at baseline and 3-months after surgery. Hereafter, a description (i.e. demographics) of the total study population follows, with a comparison between patients with and without NP (at baseline, and at 3-months after LIF). Then, the outcome-effect of LIF on the total study population is presented. These outcomes are further scrutinized by analyses that highlight the burden of disease and outcomes, based on both, NP status at baseline, and on NP status post-surgery. Finally, the long-term effect of post-surgery NP on outcomes and medication use are put forward.

### 3.1 Neuropathic pain occurrence

A total of 146 patients had NP assessments both at baseline and 3 months follow-up and were included in the analysis. At pre-op 74 patients presented a DN4 ≥ 4, whereas 72 patients presented a DN4 < 4. Based on NP occurrence at baseline and at follow-up four different groups are identified: 20 patients with persistent NP (DN4 ≥ 4 at both baseline and follow-up), 63 patients with absent NP (DN4 < 4 at both baseline and follow-up), 54 patients with resolved NP (DN4 ≥ 4 before the surgery, and DN4 < 4 at follow-up), and 9 patients with acquired NP (DN4 < 4 before the surgery and DN4 ≥ 4 at follow-up). At 3-months post-surgery, in total 29 patients presented NP, whereas 117 patients did not present NP (Table 1). Patient demographics for the total population (n = 146), and analyses based on NP status at baseline (n = 74 NP versus n = 72 no NP) and at 3 months after LIF surgery (n = 29 NP versus n = 117 no NP) were analyzed (Table 2).

**Table 1:** Occurrence of Neuropathic Pain at Baseline and at 3-months post-Surgery (N = 146).

Baseline		3-months post-surgery	
*NP*	74 (51%)	*NP*	20 (14%)^a^
		*No NP*	54 (37%)
*No NP*	72 (49%)	*NP*	9 (6%)^a^
		*No NP*	63 (43%)

NP, neuropathic pain.

a total of n = 29 patients present NP 3 months post-surgery.

**Table 2:** Baseline patient characteristics in all patients (A), differences between patients based on neuropathic pain status at baseline (B), and differences between patients based on neuropathic pain status at 3-months post-op (C).

	A: Total Study Population		B: Differences based on NP status pre-op^b^					C: Differences based on NP status at 3-months post-op^b^				
			NP at BL		No NP at BL		P-value	NP at 3M		No NP at 3M		P-value
	N = 146		n = 74		n = 72			n = 29		n = 117		
Age	58.8	±10.6	59.1	±11.2	58.5	±10.0	*0.719*	58.8	±10.5	58.8	±10.7	*0.971*
BMI	27.2	±4.1	27.3	±4.4	27.1	±3.8	*0.823*	27.3	±3.3	27.2	±4.3	*0.935*
Gender (Female %)	58.2		56.8		59.7		*0.740*	44.8		61.5		*0.140*

**Main pathology at index level**												
Stenosis	87.0%	127/146	87.8%	65/74	86.1%	62/72	*0.809*	96.6%	28/29	84.6%	99/117	*0.123*
Spondylolisthesis (grade I - II)	75.3%	110/146	73.0%	54/74	77.8%	56/72	*0.567*	62.1%	18/29	78.6%	92/117	*0.090*

**Main reason for surgery**												
Leg Pain	46.6%	68/146	51.4%	38/74	41.7%	30/72		41.4%	12/29	47.9%	56/117	
Back Pain	36.3%	53/146	29.7%	22/74	43.1%	31/72	*0*.*254*^a^	34.5%	10/29	36.8%	43/117	*0*.*494*^a^
Neurogenic Claudication	17.1%	25/146	18.9%	14/74	15.3%	11/72		24.1%	7/29	15.4%	18/117	

BL, baseline; NP, neuropathic pain.

a p-value represents the differences for the entire group considering the three options for ‘Main reason for surgery’.

b Part B and part C contain the total patient group (A), yet with different analyses based on either the NP status pre-op (B), or the NP status 3-months post-op (C).

### 3.2 Patient demographics at pre-op

The average pain duration before the spine surgery was similar between patients with predominant LP or LBP (29.1 months and 22.7 months, respectively). There were no differences at baseline in age, gender, Body Mass Index (BMI), main reason for surgery (back pain, leg pain, neurogenic claudication), main pathologies at level(s) to be operated (stenosis or spondylolisthesis), or duration of pain before surgery whether NP was present at pre-op or 3-months post-surgery when compared with the patients without NP (Table 2). Both groups with and without NP post-surgery had similar occurrence of Diabetes Mellitus (Total population: 15/146 (10.3%); NP vs no NP at 3 months post-op 3/29 (10.3%) vs 12/117 (10.3%), p = 1.000); and Peripheral Vascular Disease (Total population: 9/146 (6.2%); NP vs no NP at 3 months post-op 2/29 (6.9%) vs 7/117 (6.0%), p = 1.000).

### 3.3 Improved patient reported outcomes in the total study population after LIF

Significant improvements were observed in VAS-LP (6.14 ± 2.59 pre-op versus 1.85 ± 2.42 at 3 months post-op, p < 0.001) and VAS-LBP (6.05 ± 2.55 pre-op versus 2.81 ± 2.48 at 3 months post-op, p < 0.001) scores, and EQ-5D index (0.501 ± 0.224 pre-op versus 0.770 ± 0.165 at 3 months post-op, p < 0.001) from baseline to three months after LIF surgery in the total study population, i.e., when the analyses considered all patients, irrespective of NP occurrence.

### 3.4 Neuropathic pain at baseline: burden of disease and patient reported outcomes

#### 3.4.1 Differences at baseline

There were 51% (74/146) patients with NP at baseline. The patients presenting NP before LIF surgery had significantly higher VAS-LP (p = 0.007) and VAS-LBP (p = 0.003) scores, and lower EQ-5D index (p < 0.001) (Table 3).

**Table 3:** Changes in patient reported outcomes after surgery from baseline to 3 months based on neuropathic pain status at pre-op.

		NP at pre-op *(n=74)*		No NP at pre-op *(n=72)*		P-value
Leg Pain (VAS)	Baseline	6.70	±2.16	5.56	±2.86	*0.007*
	3 months	2.42	±2.80	1.26	±1.79	*0.005*
	p-value compared with BL	*0.002*		*<0.001*		
Back Pain (VAS)	Baseline	6.68	±2.28	5.42	±2.68	*0.003*
	3 months	2.80	±2.59	2.82	±2.38	*0.545*
	p-value compared with BL	*<0.001*		*<0.001*		
Quality of Life (EQ-5D)	Baseline	0.435	±0.217	0.570	±0.212	*<0.001*
	3 months	0.737	±0.193	0.804	±0.124	*0.099*
	p-value compared with BL	*<0.001*		*<0.001*		

BL, baseline; EQ-5D, EuroQoL 5D-3L; NP, neuropathic pain; VAS, Visual Analog Scale.

#### 3.4.2 Different response to surgery

Despite significant improvements from baseline in all groups, the VAS-LP remained significantly higher at three months post-surgery (p = 0.005) in patients with NP at baseline, whereas there was no difference in the VAS-LBP score. There was also no statistically significant difference in QoL between the groups at three months after surgery (Table 3).

### 3.5 Neuropathic pain at 3-months after LIF: burden of disease and patient reported outcomes

Of the total study population 14% (20/146) had persistent NP, and additionally 6% (9/146) had acquired NP after the fusion surgery, in total 20% (29/146) of the patients had NP after surgery (Table 1).

#### 3.5.1 Differences at baseline

There were no differences at baseline between the patients with and without NP at 3 months post-surgery regarding VAS-LP, VAS-LBP and QoL (Table 4).

**Table 4:** Changes in patient reported outcomes after surgery from baseline to 3 months based on neuropathic pain status at 3 months post-op.

		NP at 3M post-op *(n=29)*		No NP at 3M post-op *(n=117)*		P-value
Leg Pain (VAS)	Baseline	6.31	±2.77	6.09	±2.55	*0.688*
	3 months	4.00	±2.73	1.31	±2.02	*<0.001*
	p-value compared with BL	*0.002*		*<0.001*		
Back Pain (VAS)	Baseline	6.86	±2.31	5.85	±2.58	*0.057*
	3 months	4.03	±2.34	2.50	±2.43	*0.006*
	p-value compared with BL	*<0.001*		*<0.001*		
Quality of Life (EQ-5D)	Baseline	0.450	±0.212	0.514	±0.226	*0.168*
	3 months	0.659	±0.176	0.798	±0.151	*<0.001*
	p-value compared with BL	*<0.001*		*<0.001*		

BL, baseline; EQ-5D, EuroQoL 5D-3L; NP, neuropathic pain; VAS, Visual Analog Scale.

#### 3.5.2 Different response to surgery

The group of 29 patients that had NP after spine fusion responded differently to surgery, when compared with the larger group without NP at 3 months post spine fusion. The patients with NP at follow up had significantly lower VAS-LP (p < 0.001) and VAS-LBP (p = 0.006) alleviation, and lower QoL (p < 0.001) (Table 4).

### 3.6 Long-term burden of post-operative neuropathic pain on patient reported outcomes and opioid use until 1 year after LIF

**Fig. 1:**
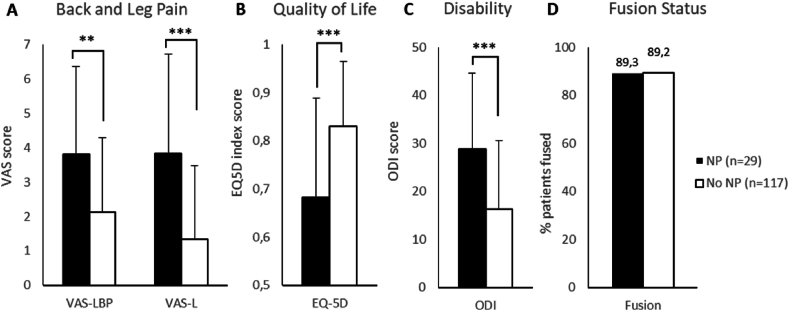
Patient reported outcomes at 1 year after surgery and fusion based on neuropathic pain status at 3 months post-op. EQ-5D, EuroQoL 5D-3L; NP, neuropathic pain; ODI, Oswestry Disability Index; VAS-L, Visual Analog Scale-Leg Pain; VAS-L, Visual Analog Scale- Low Back Pain. Data is presented as average + stdev (VAS-L, VAS-LBP, QoL and ODI or as % patients (fusion)) P-value, ∗∗<0.01, ∗∗∗<0.001.

The 29 patients, which present post-operative NP 3 months after LIF, had equal fusion success rate when compared with the group that did not present NP post-op (Fig. 1D). This NP group still presented significantly higher VAS-LP and VAS-LBP, significantly higher ODI and a lower EQ-5D score at one year after fusion surgery (Fig. 1A–C).

**Fig. 2:**
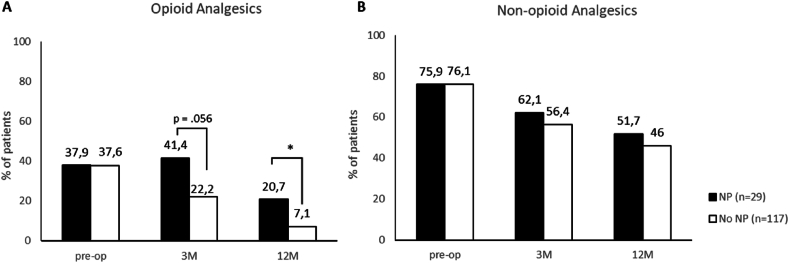
Evolution of opioid and non-opioid analgesic use after spine fusion surgery. NP, neuropathic pain; Data are presented as % of patients on medication at follow-up. P-values are based on t-test between NP and No NP groups; ∗ = p value <0.05.

Opioid analgesics consumption after surgery was higher in patients with NP when compared with the no-NP group (Fig. 2A). Consumption of non-opioid analgesics was not different between the groups (Fig. 2B).

## 4 Discussion

Lumbar interbody fusion surgery is successful in alleviating pain and improving QoL in DLD patients with chronic pain resistant to conservative treatment (Franke et al., 2016; Pereira et al., 2015a). This study represents a continuation from earlier work (i.e., MASTERS-D and MASTERS-D 2) and investigates the occurrence and role of NP in persistent pain after spine surgery and QoL in adult DLD patients treated with fusion surgery. NP was present in 51% of the DLD patients before fusion surgery and associated with higher back and leg pain scores, and lower QoL. After fusion surgery significant pain relief and improved QoL was achieved for all patients, but patients with NP at baseline had lower leg pain alleviation. Furthermore, still 1 out of 5 patients had reported NP after fusion surgery. These patients with NP at three months post-surgery had also lower leg and back pain relief and lower QoL up to 1 year after spine fusion, when compared with patients without NP. These results indicate that fusion surgery is not optimally effective in every fifth patient treated.

The results provide strong evidence that NP occurs frequently in DLD patients, and correlates with lower QoL and higher pain scores, in line with previously reported results from a similar patient population (Kim et al., 2015). Also similarly, the presence of NP at baseline did not differentiate the outcomes after spine fusion surgery (Kim et al., 2015). Persistent NP was previously reported in patients undergoing microdiscectomy, with modest to no pain relief at all (Shamji and Shcharinsky, 2016). Investigating further, our study reports for the first time significant differences in response to LIF when considering persistent and emerging NP after spine fusion surgery, despite more patients in the NP group were using opioid analgesics.

Neuropathic pain occurs along with nociceptive pain in degenerative spine patients (Kim et al., 2015; Hiyama et al., 2022). There are currently however no generally accepted algorithms for routinely identifying and addressing NP in spine surgery clinical practice, and the role of NP in persistent postoperative pain is insufficiently investigated and reported. This study identified a strong association in DLD patients between NP and the risk for lower pain alleviation and worse health status. Based on the present results it is difficult to establish the origin of NP in these spine patients, however, known chronic conditions that could induce NP, such as DM (Schreiber et al., 2015) and PVD, were excluded by our results. Neither could a difference in the fusion rates between the NP and no-NP patient groups justify a difference in reported outcomes at different time-points after surgery.

Routinely addressing NP before and after spine surgery could identify patients at risk for persistent or acquired NP during or after the spine surgery. The need to recognize patients at risk (Baber and Erdek, 2016), and a multidisciplinary team to establish criteria for appropriate treatment options (Tronnier et al., 2019) have been proposed as solutions towards improving the consistency and value of care in patients with PPSS. Spinal cord stimulation (SCS) is one such treatment option that shows promise in, at least, alleviating chronic pain in the cervical and lumbar regions (Provenzano et al., 2017). Strong evidence from large randomized clinical trials (Kumar et al., 2007; North et al., 2007) and low-to-moderate quality evidence from systematic reviews (Knotkova et al., 2021) exists for the superiority of SCS over conservative management and repeated surgery for FBSS. Furthermore, SCS appears to be an acceptable treatment for patients experiencing persistent postoperative neuropathic pain providing a potential solution for addressing NP in DLD surgery patients (Knotkova et al., 2021; Samuel et al., 2017).

Using the DN4 questionnaire, we identified the presence of NP in 51% of the patients at baseline, and in 20% at 3 months after spine fusion. These results are in line with another study from a similar patient population. Hiyama et al. (2022) used the painDETECT questionnaire and identified a NP component in 20.4% of the patients before the spine fusion surgery, while others (Kim et al., 2015) showed a 36.4% occurrence of NP when using a LANSS score. In a retrospective analysis of a large medical and pharmaceutical claims database based on ICD-9 codes (including over 55 million patients), estimates of the proportion of chronic low back pain patients with a neuropathic component was shown to range from 17 to 54% and to vary according to the method of classification used (Mehra et al., 2012b). Neuropathic pain accounted for 5% of the postoperative complications in adult spine surgery (Street et al., 2012). As many as 28% of patients had an uncertain diagnosis, possibly indicating that the neuropathic component in chronic back pain could be underestimated (Mehra et al., 2012b). Schmidt et al. (2009) suggested that a neuropathic component is more frequent in persons with severe back pain compared to persons with mild back pain.

The large variations reported could be explained by the complex causes of NP, the variability in the measurement between questionnaires or methods, and the characteristics of the DN4 questionnaire itself. NP may be caused by lesions of nociceptive sprouts within the degenerated disc (local neuropathic), mechanical compression of the nerve root (mechanical neuropathic root pain), or by action of inflammatory mediators (inflammatory neuropathic root pain) originating from the degenerative disc even without any mechanical compression. In a recent systematic review, the DN4 was identified as one of the most suitable NP screening tools for clinical use. The sensitivity of the DN4 ranges from 75 to 98%, with a moderate sensitivity for detecting a NP component in patients with chronic pain, and specificity ranging 37–96% (Mathieson et al., 2015). At cut off 4, also used in the present study, sensitivity reported ranges between 80 and 96%, while the specificity was between 6.8 and 95%. DN4 is a valuable clinical tool, providing an indication of the presence of the NP quality (Timmerman et al., 2017). However, the NP diagnosis and management remain a challenge, mainly because there is no gold standard for either (Freynhagen and Baron, 2009). Whereas the association of NP with a higher disease burden is clear within the current study, further analyses would be required to understand the implications of NP with respect to pain medication, neurological status, health care costs, and longer term follow up. It could thus be argued that NP screening questionnaires have limited measurement properties (Mathieson et al., 2015; Markman et al., 2015). However, even more important would be to address the fact that NP comorbidity in DLD patients is often not identified at all. Many spine surgery patients are treated lacking awareness of the fact that NP could lead to lower outcomes after surgery.

A Cochrane review showed moderate to weak evidence in supporting the use of opioids in treating NP (McNicol et al., 2013). In our study we showed low pain alleviation and higher disease burden up to 1 year after surgery, possibly indicating that the analgesic effect of the opioids in spine fusion patients with NP is subject to considerable uncertainty in the shorter-term follow-up. Neurostimulation (i.e. SCS) could be considered in spine fusion patients with NP and without clear indications for re-operations (Tronnier et al., 2019).

Distinguishing between chronic nociceptive pain and neuropathic pain very often remains a challenge. Clinical evaluation of NP requires a thorough history and physical examination to identify characteristic signs and symptoms which should be considered in future spine studies.

### 4.1 Strengths and limitations

We report clinical evidence from a large, multi-center, prospective, cohort study, with appropriate statistical methods and validated questionnaires, assuring for high quality, and transparency of the evidence. Clinically relevant and statistically significant data on the role of NP in insufficient pain alleviation after spine fusion surgery was presented.

The main purpose of using the DN4 questionnaire was to identify the presence of a NP component in this patient population, rather than to optimally assess the quality and intensity of the NP.

The changes in NP status are for the first time analyzed both prospectively from baseline and retrospectively at follow up, indicating changing NP status and that spine surgery in itself could inflict NP symptoms.

We preferred the DN4 questionnaire in this study as a screening tool for neuropathic pain due to its ease of administration. As described, the cut-off scoring at 4 or more points was used, to identify the presence of a NP component. Future studies could evaluate whether the absolute score correlates with patient outcomes, potentially classifying mild-NP versus intense-NP group which could further improve patient triage.

Furthermore, other NP screening tools and a multi-team approach with physician bedside anamnesis and laboratory tests could further improve the reliability of the NP assessment.

EC/IRB approvals to administer the DN4 questionnaire prospectively in this study were received after study start which explains why not all DLD patients were assessed for NP.

A randomized controlled trial in a larger number of patients could have been used to minimize confounding factors due to possible differences in the standard of care between sites and offer a higher level of statistical probability.

This study reports on increased opioid use in the NP group, with unsatisfactory results in pain decrease, aiming to highlight a relevant issue with regards to opioid overconsumption. In future, evaluation of nerve pain medication, such as gabapentin and/or pregabalin, could be investigated to evaluate their effect on patients suffering from NP.

With the present study we confirm for the first time that NP in DLD patients represents a common phenomenon, leading to higher opioid consumption, and occurring independently of known NP triggers such as DM or PVD.

## 5 Conclusions

Neuropathic pain occurred frequently in DLD patients, both before and after spine fusion surgery. In patients with post-operative NP there was a significant association between NP, lower pain alleviation and higher disease burden up to 12 months post-op, in spite of higher opioid consumption. NP occurred independently of DM, PVD, and fusion success. Pre- and post-operative assessments for NP via a multi-team approach should be routinely performed for timely diagnosis and treatment of NP in spine fusion patients, for improved patient outcomes and healthcare consumption.

## Disclosures

SW Park, C Santos, and V Arzoglou declare that they have nothing to disclose; A Krutko received a research grant from Medtronic; W Senker has a consultancy with Medtronic, P Pereira has a consultancy and education agreement with Medtronic. The authors had final responsibility for the content of the manuscript and decision to submit for publication and were not paid to write the article by Medtronic or any other agency.

## Patient consent forms and IRB/research ethics committee approvals

If required by local regulations, IRB and/or ethics approval was obtained prior to patient recruitment and administration of any study procedure. Informed consent was given by patients prior to any study-related procedure.

## Funding statement

This study was funded by 10.13039/100031395Medtronic Bakken Research Center, The Netherlands. Medtronic's Marketing and Sales division was not involved in the development of this study, analysis, or interpretation of the data, writing of the manuscript, or decision to submit for publication.

## Declaration of competing interest

The authors declare the following financial interests/personal relationships which may be considered as potential competing interests: Seung Won Park, Aleksandr Krutko, Carlos Santos, Wolfgang Senker, Vasileios Arzoglou and Paulo Pereira reports financial support, administrative support, and statistical analysis were provided by Medtronic Bakken Research Center BV. Aleksandr Krutko reports a relationship with Medtronic Cranial and Spinal Technologies that includes: funding grants. Wolfgang Senker reports a relationship with Medtronic Bakken Research Center BV that includes: consulting or advisory. Paulo Pereira reports a relationship with Medtronic Bakken Research Center BV that includes: consulting or advisory. B Heijnen is employed within Clinical Affairs, Cranial and Spinal Technologies, Medtronic. When the work was performed on this manuscript A Cristea was employed at the Office of Medical Affairs, Cranial and Spinal Technologies, Medtronic. If there are other authors, they declare that they have no known competing financial interests or personal relationships that could have appeared to influence the work reported in this paper.
