# Supplementary material for: Neuropathic pain appears to be the main symptom associated with higher disease burden and lower pain alleviation in degenerative lumbar disease fusion patients
Source: Brain Spine. 2025 Feb 25;5:104224. doi: 10.1016/j.bas.2025.104224 (PMC11925177; doi:10.1016/j.bas.2025.104224)
Supplement: Multimedia component 1 [file mmc1.docx]

*‘Neuropathic Pain Appears to be the Main Symptom Associated with Higher Disease Burden and Lower Pain Alleviation in Degenerative Lumbar Disease Fusion Patients’*

**DN4 study cohort (n = 146)**

To answer our research questions the NP status both at baseline and 3M follow-up is required. Patients were followed up until 12 months after fusion surgery.

## 12 months

Excluded (n = 25)

♦  Not treated with MIS fusion as per protocol (n = 20)

♦  Declined to participate (n = 1)

♦  Other reasons (n = 4)

*** IMPORTANT NOTE**
the DN4 was introduced into this study when it was already enrolling. Hence, not all patients could be administered to the questionnaire at baseline and 3M follow-up timepoints. After EC/IRB submissions for the administration of DN4, patients were assessed for NP until the patient recruitment ended. Patients for which the NP status could not be determined based on the DN4 assessments, were excluded from analysis.

Excluded from analysis due that DN4 for either leg or

back is missing and patient NP status could not be

determined (partial DN4 assessment) (n = 12)

Patients missing DN4 assessments for leg and back at

baseline according to protocol (n = 13)

Patients missing DN4 assessments for leg and back at

3 months according to protocol (n = 23)

Patients not eligible for DN4 at baseline (n = 146)

Reason see* IMPORTANT NOTE

## Enrollment

## 3 months

## Baseline

Patients with DN4 at baseline
**AND** at 3 months (n = 158)

Patients eligible for DN4 at baseline (n = 194)

Eligible DLD patients entering the MASTERS-D 2 study (n = 365)

Eligible DLD patients treated per protocol (n = 340)

## Analysis
